# Analysis and identification of volatile aroma compounds of the garlic-scented mushroom *Mycetinis scorodonius*

**DOI:** 10.1016/j.fochx.2026.104033

**Published:** 2026-05-27

**Authors:** Jenny Ahlborn, Deria Yusein, Christoph Hartwig, Tatyana Zhuk, Lea-Angel Emrich, Annika E.L. Beiderwieden, Florian Birk, Andreas K. Hammer, Anne Steinkamp

**Affiliations:** a Institute of Food Chemistry and Food Biotechnology, Justus Liebig University Giessen, Heinrich-Buff-Ring 17, 35392 Giessen, Germany; b Fraunhofer Institute for Molecular Biology and Applied Ecology (IME), Branch for Bioresources, Ohlebergsweg 12, 35392 Giessen, Germany; c Faculty of Chemical Technology, Igor Sikorsky Kyiv Polytechnic Institute, Beresteiskyi Ave, 37, Kyiv 03056, Ukraine

**Keywords:** Fruiting bodies, Methyl dithioformate, Marasmicin, Aroma profile, *Tulbaghia violacea*

## Abstract

Fruiting bodies of *Mycetinis scorodonius*, a mushroom with an intense garlic flavor, were cultivated under laboratory conditions. Focusing on sulfur compounds, volatile organic compounds of the freeze-dried fruiting bodies were analyzed for the first time by means of headspace solid-phase micro extraction and GC–MS/MS-olfactometry. Nine sulfur compounds were identified, including primarily methanethiol, methyl dithioformate, 2,3,5-trithiahexane, dimethyl trithiocarbonate and 2,4,5,7-tetrathiaoctane. For the first time, methyl dithioformate was detected in *Mycetinis* species. Only four aroma compounds were found in a dichloromethane extract, which were also quantitated. For comparison, the garlic-scented plant *Tulbaghia violacea* was extracted due to the presence of the thiosulfinate marasmicin. Measurements of liquid *Mycetinis* and *T. violacea* extracts by UHPLC-MS and GC–MS/MS-O indicated the presence of the aroma precursor marasmicin in the fungal extract. GC–MS/MS measurements of a pure HPLC marasmicin fraction revealed the thermal degradation products of marasmicin.

## Highlights

• Laboratory cultivation of the garlic-scented mushroom *Mycetinis scorodonius*.
• Aroma profile analysis using HS-SPME GC–MS/MS revealed 9 sulfur compounds.
• First time identification of the aroma compound methyl dithioformate in fungi.
• Precursor molecule marasmicin was detected in the liquid extract using HPLC.
• Quantitation of aroma compounds in the extract showed 631 mg/kg 2,3,5-trithiahexane.

## 1 Introduction

The great variety of mushrooms is known for their partly unique flavors, such as *Laetiporus sulphureus* (chicken of the woods), *Lentinellus cochleatus* (aniseed cockleshell) or mushrooms from the genus *Mycetinis* (Rapior, Breheret, Talou, & Bessière, 1997; Rapior, Breheret, Talou, Pélissier, & Bessière, 2002; Yalman et al., 2023). *Mycetinis scorodonius* (formerly known as *Marasmius scorodonius*) is a mushroom with small fruiting bodies that have an intense garlic-like smell and taste, especially when wet or cells were crushed (Gmelin, Luxa, Roth, & Höfle, 1976). The name is derived from the Greek word *skórdo* (σκόρδο) for garlic. The fungus is native to large parts of Central Europe and forms fruiting bodies in summer and late summer until November (Breitenbach & Kränzlin, 1991; Laux, 2019). Due to their unique aroma, *M. scorodonius* or its relative *M. alliaceus* are attractive spices (Rapior et al., 1997). As a seasoning mushroom, it has a wide range of uses and can be eaten raw or cooked. It is said that eating the garlic-scented mushrooms, unlike garlic, does not cause any unpleasant breath or body odor due to different aroma compounds, but no studies have been published to date. There is no industrial production until today, as the fruiting bodies are very thin and cultivation is difficult due to specific requirements for temperature, humidity, and light. The fruiting bodies of *M. scorodonius* can easily be dried without any considerable loss of flavor, which makes them interesting for storage and sale (Laux, 2019).

**Fig. 1:**
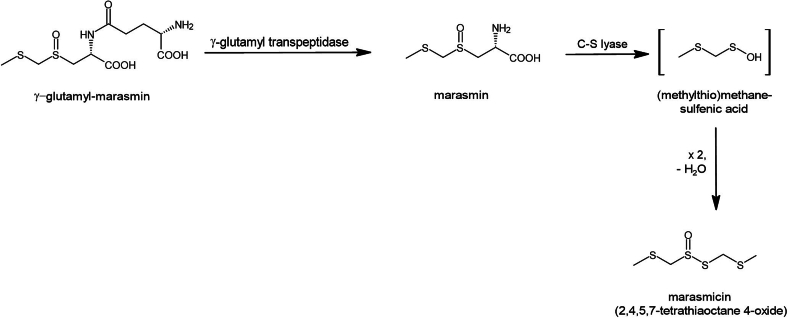
Formation of the aroma intermediate 2,4,5,7-tetrathiaoctane 4-oxide (marasmicin) in *Mycetinis* species according to Gmelin et al.^4^

Aliphatic C_8_ hydrocarbons, ketones and alcohols such as oct-1-en-3-one, oct-1-en-3-ol and octan-3-one are primarily responsible for the typical mushroom-like aroma (Karrer, Weigel, Hoberg, Atamasov, & Rühl, 2021; Marcinkowska & Jeleń, 2022; Matsui, Sasahara, Akakabe, & Kajiwara, 2003). However, sulfur-containing compounds also play an important role in some mushrooms, such as shiitake, truffles or *Mycetinis* (Mustafa et al., 2020; Rapior et al., 1997; Schmidberger & Schieberle, 2020). So far, only a few publications focused on the sulfur-containing aroma precursors of *Mycetinis* species and their formation pathways. Gmelin et al. showed that the origin of the flavors is the dipeptide *γ*-glutamyl-marasmin, which is cleaved by a *γ*-glutamyl transpeptidase to marasmin, a cysteine sulfoxide derivative (CSO; Fig. 1) (Gmelin et al., 1976). A C-S lyase produces (methylthio)methanesulfenic acid, which condenses to thiosulfinate 2,4,5,7-tetrathiaoctane 4-oxide (synonym marasmicin (Kubec, Krejčová, Mansur, & García, 2013)). Marasmicin is subjected to spontaneous disproportionation, during which many sulfur-containing aroma compounds are released (Gmelin et al., 1976). This enzymatic pathway is the counterpart to the reactions that take place in garlic (*Allium sativum*) and are well examined: after the disruption of the garlic cells, the enzyme alliinase stored in the vacuole reacts with alliin (*S*-allyl-l-cysteine-S-oxid) stored in the cytoplasm producing 2-propenesulfenic acid, which condenses forming the thiosulfinate allicin (diallyl thiosulfinate) (Kubec et al., 2013). Allicin is the key molecule in garlic flavor and represents about 70% of the total thiosulfinates that are produced when a garlic clove is cut (Marcinkowska & Jeleń, 2022). Due to its instability, it can easily be converted into other flavors such as diallyl mono-, di-, tri- and tetra-sulfides.

The stereochemistry of *γ*-glutamyl-marasmin in fungi (*S*_S_,*R*_C_) differs from that of marasmin found in plants (*R*_S_,*R*_C_) like *Tulbaghia violacea* (known as society garlic) or different *Allium* species (Kubec et al., 2011; Kubec, Velíšek, & Musah, 2002; Kusterer, Fritsch, & Keusgen, 2011). Kusterer et al. analyzed the CSO amount in 74 different *Allium* samples (up to 2.25% total CSO amount, related to the fresh weight of bulbs) and also two samples of the fungus *M. alliaceus* (Kusterer et al., 2011). In fungal samples, up to 2.99% of *γ*-glutamyl-marasmin are present (related to air-dried fruiting bodies), but no further CSO were detected (Kusterer et al., 2011). Plants such as *T. violacea* have long been known for their antimicrobial effect. Marasmicin is biologically active, has antimicrobial and antifungal effects and can be used against various microbial infections (Kusterer et al., 2011; Ranglová, Krejčová, & Kubec, 2015). Marasmicin not only has an antimicrobial effect, but is also the precursor for the garlicky aromas of *T. violacea* and *Mycetinis* species (Gmelin et al., 1976; Kubec et al., 2013; Rapior et al., 1997). Up to now, there have been very few studies focusing on the aroma profile of *Mycetinis* species. Rapior et al. investigated the volatile aroma compounds of *M. alliaceus* using both solvent extraction and dynamic headspace concentration (Rapior et al., 1997). They have found various sulfur-containing compounds, including 1,3-dithiethane, 2,3,5-trithiahexane and 2,4,5,7-tetrathiaoctane.

This work was aimed at investigating and identifying the sulfur-containing volatile aroma compounds of *M. scorodonius* for the first time. Mushrooms were cultivated under laboratory conditions and analyzed for their aroma profile and flavor dilution factors by means of headspace solid-phase micro extraction gas chromatography coupled with tandem mass spectrometry and olfactometry (HS-SPME GC–MS/MS-O). In addition, a dichloromethane extract was examined and the aroma compounds found were quantitated.

## 2 Materials and methods

### 2.1 Chemicals

Cultivation media were purchased from Th. Geyer (Renningen, Germany) and Carl Roth (Karlsruhe, Germany). The grain for fruiting body production was provided by LWB Hirt (Weißensee, Germany). Authentic standards were used for identification of the sulfur-containing aroma compounds: 2,3,5-trithiahexane (>91%) was purchased from Smolecules (San Antonio, USA), dimethyl trisulfide (>98%) and 2,4-dithiapentane (>98%) from TCI (Eschborn, Germany), carbon disulfide (99.9%), diallyl disulfide (>95%) and dimethyl trithiocarbonate (98%) from Sigma Aldrich (Taufkirchen, Germany), methanethiol (1 mg/mL) from Biozol (Eching, Germany) and dimethyl disulfide (99%) from Thermo Fisher Scientific (Schwerte, Germany). In addition, four C_8_-aroma compounds were used for identification: octan-3-one (98%) was obtained from Alfa Aesar (Karlsruhe, Germany), oct-1-en-3-on (>96%) from Sigma Aldrich, oct-1-en-3-ol (98%) and dl-octan-3-ol (97%) from Acros Organics (Geel, Belgium). Dichloromethane, acetonitrile (both HPLC grade; Th. Geyer, Renningen, Germany) and sodium sulfate (99%, anhydrous; Thermo Fisher Scientific) were used for fruiting body extraction. For methyl dithioformate synthesis, super-hydride solution (1.0 M lithium triethylborohydride in THF), iodomethane (99%) from Sigma Aldrich and tetrahydrofuran (99.5%, extra dry) from Thermo Scientific were used. 1,3-Dithiethane was produced using bis(chloromethyl)sulfide (>97%) from TCI. The plant *Tulbaghia violacea* was purchased from Blu-Blumen GbR (Langenberg, Germany).

Due to the strong odor and the toxicity of dimethyl disulfide, all aroma standards were only opened under the fume hood. The synthesis of methyl dithioformate was carried out under the fume hood due to the toxicity of iodomethane and the harmful effects of the solvents.

### 2.2 Cultivation of fungi and sample preparation

*Mycetinis scorodonius* was purchased from Steintaler Edelpilz (Neu Wulmstorf, Germany). For strain maintenance, *M. scorodonius* was cultivated on malt extract agar plates (2% malt extract and 1.5% agar-agar). The preculture was prepared as described by Trapp et al. (Trapp et al., 2018): 1 cm^2^ of the overgrown agar plate was placed in a sterile 250 mL Erlenmeyer flask containing 100 mL of a 2% malt extract solution. After homogenization by means of an Ultra-Turrax T25 homogenizer (IKA, Staufen, Germany) for 30 s at 10,000 rpm, the pre-culture was cultivated on a rotary shaker (Orbitron, Infors, Einsbach, Germany) at 24 °C and 150 rpm for 9 days in darkness. After homogenization of the pre-culture, the suspension was used to inoculate agricultural products such as oats (*Avena sativa*) as substrates for fruiting body production. The fungi were cultivated in plastic boxes on the laboratory bench at room temperature (21–22 °C), with high humidity (> 90% RH) and CO_2_ levels below 1000 ppm.

For sample preparation, mature fruiting bodies from several batches were freeze-dried, combined, and ground, passed through an analytical sieve of 0.2 mm mesh and stored at −20 °C until further analysis.

### 2.3 Headspace solid phase microextraction (HS-SPME)

For HS-SPME analysis, 100 mg dried fruit bodies and 3 mL water were weighed into 20 mL headspace vials, sealed with silicon/PTFE screw caps. The samples were incubated for 10 min at 40 °C and 250 rpm using an MPS multipurpose sampler (Gerstel, Mülheim an der Ruhr, Germany). Extraction was performed using DVB/C-WR/PDMS (80 μm) SPME fiber (Supelco, Steinheim, Germany) for 30 min under the same conditions, unless otherwise mentioned.

### 2.4 Liquid extraction

Following an extraction method for marasmicin (Kubec et al., 2013) with minor modifications, dried *M. scorodonius* fruiting bodies were extracted with dichloromethane. The procedure was as follows: 5 g of freeze-dried and ground fruiting bodies were mixed with 108 mL of water to activate the enzymatic cascade leading to the formation of marasmicin. In case of quantitation 400 μL of internal standard diallyl disulfide (2 mg/mL stock solution in ethanol) were added. The mixture was kept at room temperature for 30 min. After filtering through a pleated filter, the liquid phase was extracted twice with 67 mL dichloromethane. Combined organic layers were centrifuged (3680 *g*, 4 °C, 10 min), dried over sodium sulfate and concentrated to dryness in a rotary evaporator at 35 °C and 650 mbar. The residue was redissolved in acetonitrile and filtered through a 0.2 μm syringe filter. This extract was analyzed using GC–MS/MS-O and UHPLC-HR-MS. For *T. violacea*, approx. 10 g of fresh leaves of the plant was cut into small pieces and homogenized together with water using a mixing rod (MultiQuick 5 Vario; Braun, Neu-Isenburg, Germany). Extraction was performed as described above.

### 2.5 Gas chromatography analysis

HS-SPME gas chromatography (GC–MS/MS-O, scan mode in Q1) was performed as described by Trapp et al. (Trapp et al., 2018) on a polar column (Agilent J&W VF-WAXms, 30 m × 0.25 mm i.d., 0.25 μm) with a split ratio of 2:1, an inlet temperature of 250 °C and the following temperature program: 40 °C for 3 min, heating with 5 °C/min to 240 °C for 12 min. The same method was used for the analysis of liquid samples, with the appropriate liquid liner and a split ratio of 20:1.

To determine the retention indices on a non-polar column (Agilent J&W HP-5 ms Ultra Inert; 30 m × 0.25 mm i.d., 0.25 *μ*m), an Agilent 7890 A GC system equipped with a 5975C mass spectrometry detector (Agilent Technologies) was used (GC–MS). The following conditions were applied: helium as carrier gas (constant flow rate, 1.2 mL/min); inlet temperature, 250 °C; septum purge flow rate, 3 mL/min; split ratio 5:1; scan mode, TIC; scan range, *m/z* 33–300; electron ionization energy, 70 eV; source temperature, 230 °C; quadrupole temperature, 150 °C; transfer line temperature, 250 °C; temperature program, 40 °C for 3 min, heating with 5 °C/min to 320 °C for 8 min.

An Agilent 7890 A gas chromatograph coupled with a G4513A autosampler, a flame ionization detector, and an olfactory detection port (ODP 3; Gerstel) was used for cold-on-column injections. The separation was also performed on a polar VF-WAXms column using the same temperature program as described above (initial temperature: 40 °C) with an injection volume of 1 μL, 2.2 mL/min H_2_ as carrier gas (constant flow), 3 mL/min septum purge flow rate, thermal Aux 1 250 °C and Aux 2 150 °C. The FID used N_2_ as make-up gas (25 mL/min) at 250 °C, 40 mL/min H_2_, and 400 mL/min air.

### 2.6 Quantitation of odorants in the liquid extract

An internal standard (diallyl disulfide) was used to quantitate the aroma compounds in the liquid extract. Diallyl disulfide is a sulfur compound found in garlic but is not present in *M. scorodonius*, which makes it a suitable internal standard. For quantitation of 2,3,5-trithiahexane, the response factor was determined using the GC–MS/MS method for liquid samples as described under 2.5 in the range from 2 to 200 μg/mL 2,3,5-trithiahexane and 40 μg/mL internal standard (response factor: 1.29 with a correlation coefficient of 0.9965). The other sulfur compounds in the extract were semi-quantitated due to the lack of standards. In the case of 2,3,5-trithiahexane, the limit of detection was 0.5 ng and the limit of quantitation was 1.18 ng (determined using linear six-point calibration between 0.2 and 6.25 μg/mL; correlation coefficient: 0.9980).

### 2.7 Aroma dilution analysis

According to the published method of HS-SPME Aroma Dilution Analysis (ADA) (Trapp et al., 2018), dried mushroom samples were analyzed by three female people using a polar column in a GC–MS/MS-O system. The following split ratios were applied: splitless, 1:1, 3:1, 7:1, 15:1, 31:1, 63:1, 127:1, and 255:1, resulting in flavor dilution (FD) factors of 1, 2, 4, 8, 16, 32, 64, 128 and 256, respectively. The FD factors of the substances are reported as the median values.

### 2.8 UHPLC-HR-MS analysis and microfractionation

Ultrahigh performance liquid chromatography was performed using a quadrupole time-of-flight high-resolution mass spectrometry (UHPLC-HR-MS) with a C18 column and a gradient of 0.1% formic acid in water and 0.1% formic acid in acetonitrile, and both the UHPLC separation and the HR-MS parameters were carried out according to the protocol described by Eichberg et al. (Eichberg et al., 2024). For microfractionation of the liquid extract, the entire flow was collected directly after the diode array detector (DAD) in a 96-well plate with a fraction length of 16 s. Fractionation was performed twice with 10 × 5 μL. The putative marasmicin fraction and the 2,3,5-trithiahexane fraction, as well as blank fractions, were then measured using gas chromatography as described under 2.5 (polar VF-WAXms column).

### 2.9 Synthesis of methyl dithioformate and 1,3-dithiethane

Methyl dithioformate was synthesized according to the procedure described by Sakamaki et al. using carbon disulfide, 1.0 M lithium triethylborohydride in THF and iodomethane (Sakamaki et al., 2018). An aliquot was taken for nuclear magnetic resonance analysis (NMR). For GC–MS/MS-O analysis, *n*-pentane was added to the solution, which was filtered through cotton to remove inorganic salts. Formation of methyl dithioformate was confirmed by GC–MS/MS-O (purity: 34%; *m/z* (relative intensity %) 92 (100, M), 45 (26), 77 (16), 94 (10), 76 (7)) and ^1^H NMR (400 MHz; *δ* 2.52 (s, 3H) and 11.32 (s, 1H)). 1,3-Dithiethane was synthesized according to Block et al. from bis(chloromethyl)sulfide (Block, Corey, Penn, Renken, & Sherwin, 1976) and showed an MS spectrum with *m/z* 92 (100, M), 46 (26), 45 (22), 94 (10), 64 (6), 77 (1), as well as an ^1^H NMR signal at *δ* 4.04 (s, 2H). The purity of 1,3-dithiethane was 95% (GC–MS).

### 2.10 Compound identification

For compound identification, the available standards (STD) were measured on a polar and a non-polar column using GC–MS/MS-olfactometry (O) and GC–MS respectively. Retention indices (RI) were calculated according to van Den Dool and Kratz (van Den Dool & Kratz, 1963) and mass spectra (MS) were compared to authentic standards, literature and the NIST database Version 2.4. For HS-SPME measurements, water blanks were measured, and in the case of liquid extracts, solvent blanks were measured to identify peaks that did not originate from the sample.

## 3 Results and discussion

### 3.1 Fruiting bodies of *M.* s*corodonius*

**Fig. 2:**
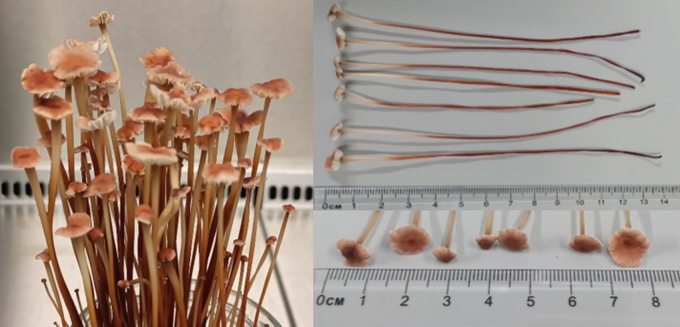
Mature fruiting bodies of *M. scorodonius* grown under laboratory conditions.

First fruiting bodies formed approximately six weeks after inoculation of the substrate. They grew up to 14 cm long, with a cap diameter of approx. 1 cm and a stalk diameter of approx. 0.2 cm. The color of the fruiting bodies varied between beige-brown and dark brown, being almost black at the bottom. The dark brown color spread from the base of the mushroom partly over the whole mushroom. Mature fruiting bodies (Fig. 2) had upwards curled caps and were able to release spores. With a length of 2.5 to 5.0 cm, the stems of mushrooms growing in nature are considerably shorter than those cultivated under laboratory conditions (Breitenbach & Kränzlin, 1991). This could be due to the environmental conditions (substrate, light, CO_2_, temperature, pH) on the one hand and the strain used on the other.

The aroma when chewing *M. scorodonius* is unique and strongly reminiscent of garlic, although it differs from the typical garlic smell in characteristic nuances. In case of *T. violacea*, a rhizomatous, grass-like plant containing the same aroma precursor as *Mycetinis* species and having an intense garlicky taste, the garlic breath lasts for several hours after consumption and therefore does not really deserve the name “society garlic” or “bad-breath-free garlic” (Kubec et al., 2013; Ranglová et al., 2015). It remains to be clarified whether the consumption of *M. scorodonius* really does not lead to any unpleasant breath and body odors of the consumers.

### 3.2 Volatile organic compounds of *M.* s*corodonius* fruiting bodies

**Fig. 3:**
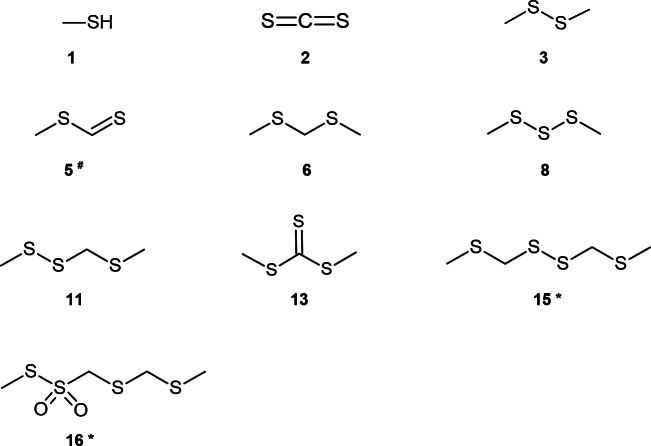
Structures of identified sulfur compounds in *Mycetinis scorodonius* fruiting bodies. Structure 16 could only be detected in the liquid extract. ^#^ identified within this work after synthesis of the compound; * tentatively identified by RI and MS.

As sulfur compounds are responsible for garlic-, onion- or cabbage-like odors (Kubec et al., 2013; Kusterer et al., 2011), the focus was placed on sulfur-containing volatile organic compounds (VOCs) in addition to C_8_ aromas when evaluating the GC–MS/MS chromatograms. A total number of 15 compounds (eleven sulfur compounds, four C_8_ compounds; Table 1; molecular masses and mass spectral data are shown in supporting information Table S1) was detected in freeze-dried fruiting bodies by means of HS-SPME GC–MS/MS-O. The three most dominant perceptible peaks were methyl dithioformate (5), oct-1-en-3-one (7) and 2,3,5-trithiahexane (11). The structures of the nine identified sulfur compounds are shown in Fig. 3. In addition, two further, unidentified sulfur compounds were detected by GC–MS/MS on the basis of the sulfur isotope ratio and the MS match factors. Both could not be detected by olfactory means and the match factors were below a value of 750, which is only a fair match according to NIST guidelines. The first unidentified peak (12) is a substance with three sulfur atoms, whereas the second unidentified peak (14) has four sulfur atoms (MS spectra are shown in supporting information **Fig. S1**). However, all odor-perceptible aroma compounds could be clearly identified.

**Table 1:** Volatile organic C_8_ and sulfur containing compounds of freeze-dried *Mycetinis scorodonius* fruiting bodies, analyzed by HS-SPME GC–MS/MS-O. Identification by retention index (RI), mass spectrum (MS), olfactometry (O) and/or authentic standard (STD); nd: not detected, na: not available.

					RI polar		RI nonpolar	
no.	compound	perceived odor	odor from literature	identification	sample	literature	sample	literature
1	methanethiol	sulfuric, putrid	rotten, sulfuric, cabbage-like^1,2^	MS, O, STD	< 900	774^1^	< 800	464^3^
2	carbon disulfide	sulfuric, putrid	radishes^4^	MS, O, STD	< 900	745^5^	< 800	544^6^
3	dimethyl disulfide	nd	sulfuric, cabbage-like, putrid^2^	RI, MS, STD	1062	1065^7^	< 800	745^8^
4	octan-3-one	etheric	earthy^9^	RI, MS, O, STD	1250	1244^10^	988	970^10^
5^#^	methyl dithioformate	garlic, sulfurous	garlic, sulfurous^1^	RI, MS, O, STD	1253	1273^1^	< 800	na
6	2,4-dithiapentane	cabbage-like, sulfuric, putrid	sulfuric, garlic-like^1^	RI, MS, O, STD	1277	1272^11^	882	892^12^
7	oct-1-en-3-one	mushroom-like	mushroom-like^13^	RI, MS, O, STD	1293	1293^10^	977^13^	976^13^
8	dimethyl trisulfide	cabbage-like, galic-like	sulfuric, cabbage-like^13^	RI, MS, O, STD	1368	1374^13^	964	972^8^
9	octan-3-ol	nd	oily, wild sesame, herbaceous^14^	RI, MS, STD	1390	1389^10^	999	988^10^
10	oct-1-en-3-ol	mushroom-like	mushroom-like^13^	RI, MS, O, STD	1442	1448^10^	978	973^10^
11	2,3,5-trithiahexane	aged garlic, savory, sulfurous	(long-standing) garlic, sulfurous, onion^15,16^	RI, MS, O, STD	1648	1654^1^	1126	1139^17^
12	not identified	nd			1734		1096	
13	dimethyl trithiocarbonate	nd	na	RI, MS, STD	1772	na	1186	1196^18^
14	not identified	nd			1984		1362	
15*	2,4,5,7-tetrathiaoctane	nd	garlic, rotten, onion with irri-tating effect^1,16^	RI, MS	2264	2278^1^	1513	1528^19^

^#^ identified within this work after synthesis of the compound; * tentatively identified; ^1^Sakamaki et al., 2018; ^2^Flaig, Qi, Wei, Yang, & Schieberle, 2020; ^3^Bonaïti, Irlinger, Spinnler, & Engel, 2005; ^4^Davidson & Feinleib, 1972; ^5^Mallia, Fernández-García, & Olivier Bosset, 2005; ^6^Insausti, Goñi, Petri, Gorraiz, & Beriain, 2005; ^7^Pozo-Bayón, Ruíz-Rodríguez, Pernin, & Cayot, 2007; ^8^Kubec et al., 2013; ^9^Kubíčková & Grosch, 1997; ^10^Wu, Zorn, Krings, & Berger, 2005; ^11^Mahadevan & Farmer, 2006; ^12^Mahattanatawee, Perez-Cacho, Davenport, & Rouseff, 2007; ^13^Schmidberger & Schieberle, 2020; ^14^Lee, Shin, & Oh, 2008; ^15^Trapp et al., 2018; ^16^Kubota & Kobayashi, 1994; ^17^Rapior et al., 1997; ^18^NIST Chemistry WebBook, SRD 69 (2026a); ^19^NIST Chemistry WebBook, SRD 69 (2026b).

In comparison with the aroma profile of *M. alliaceus*, analyzed by Rapior et al. using dynamic headspace extraction (Rapior et al., 2002), four of the nine sulfur compounds reported for *M. alliaceus* could also be detected in *M. scorodonius*: dimethyl disulfide (3), 2,4-dithiapentane (6), dimethyl trisulfide (8) and 2,3,5-trithiahexane (11). Literature data on various garlic-scented plants (e.g. wood garlic, Asian skunk cabbage, *T. violacea*) consistently report the same aroma compounds identified in *M. scorodonius*, particularly 2,3,5-trithiahexane (11) and 2,4,5,7-tetrathiaoctane (15) (Kubec et al., 2013; Kubota & Kobayashi, 1994; Sakamaki et al., 2018). This could be due to the same aroma precursor marasmin. In mushrooms, marasmin must first be released from *γ*-glutamyl-marasmin by a *γ*-glutamyl transpeptidase (Fig. 1). According to Gmelin et al., marasmin is transformed to marasmicin, the 2,4,5,7-tetrathiaoctane 4-oxide (Fig. 1) (Gmelin et al., 1976). The loss of oxygen from marasmicin leads to 2,4,5,7-tetrathiaoctane (15) and the additional loss of MeS yields 2,3,5-trithiahexane (11).

In addition to sulfur compounds, C_8_ compounds also play an important role in mushrooms. These C_8_ compounds in fungi derive from the oxidative degradation of fatty acids (e.g. linoleic acid) and associated enzyme activity (Combet et al., 2006; Politowicz et al., 2018; Schmidberger & Schieberle, 2020; L. Zhang, Zhang, & Mujumdar, 2021). Karrer et al. investigated the C_8_-oxylipin pathway in Basidiomycota and proposed a pathway that leads from linoleic acid via the activity of lipoxygenases, dioxygenases and putative hydroperoxide lyases to two biocatalytic cycles: the ketonic cycle and the aldehydic cycle (Karrer et al., 2021). The C_8_ compounds found in *M. scorodonius* all belong to the ketonic cycle. They can be converted into each other by ene-reductases, alcohol-dehydrogenases and double bond reductases (Karrer et al., 2021). Further research will determine which enzymes exactly are involved in aroma formation of *M. scorodonius* and which compounds trigger and enhance aroma production.

### 3.3 Identification of methyl dithioformate

**Fig. 4:**
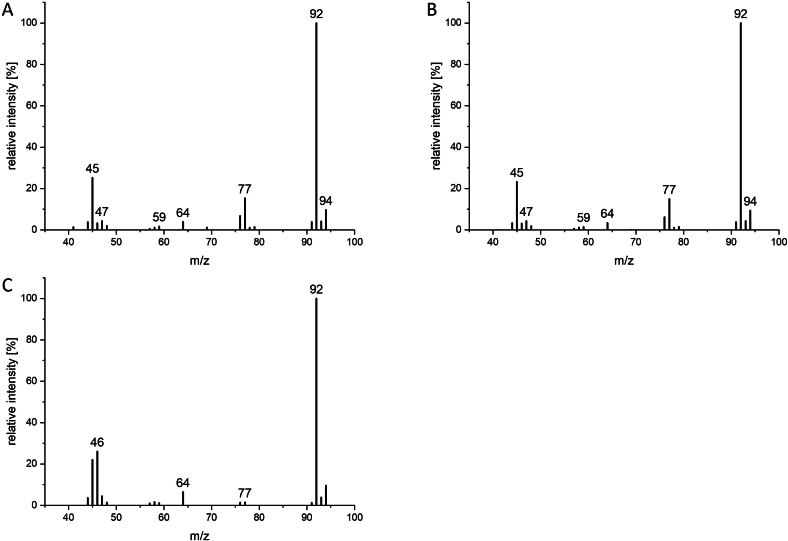
GC–MS/MS spectra (scan mode in Q1) of synthesized methyl dithioformate standard (A), odorant no. 5 from *M. scorodonius* fruiting bodies (B) and synthesized 1,3-dithiethane standard (C).

Odorant no. 5 has a very intense garlicky, sulfurous smell. The MS spectrum (*m/z* (relative intensity %) 92 (100), 45 (31), 77 (17), 94 (10), 76 (7)) was very similar to the spectrum of 1,3-dithiethane in *M. alliaceus* published by Rapior et al. (*m/z* (%) 92 (100), 45 (47), 77 (22), 94 (9), 47 (9)) (Rapior et al., 1997). However, the synthesis of 1,3-dithiethane according to Block et al. (Block et al., 1976) followed by spiking it to a *M. scorodonius* sample (see supporting information **Fig. S2**) led to two separated peaks and different odor impressions. 1,3-Dithiethane had an onion and sulfurous smell, but not a garlic-like one. Further research led to the spectrum of methyl dithioformate, which was detected by GC–MS-O for the first time in the Asian skunk cabbage in 2018 (Sakamaki et al., 2018). Synthesizing methyl dithioformate for this study and spiking a *M. scorodonius* extract with the standard resulted in a significantly increased intensity of odorant no. 5 (see supporting information **Fig. S3**) and no different odor impressions. The identification of odorant no. 5 as methyl dithioformate is also supported by MS spectra and ^1^H NMR. Fig. 4 shows that the GC–MS/MS spectra of 1,3-dithiethane and methyl dithioformate (5) differ and that the spectrum of methyl dithioformate (5) matches that of the fungal compound. ^1^H NMR results of methyl dithioformate (5; *δ* 2.52 (s, 3H) and 11.32 (s, 1H)) are in good accordance with those of Gandhi et al. (*δ* 2.68 and *δ* 11.33) and Sakamaki et al. (*δ* 2.47 and *δ* 11.29) (Gandhi, Nethaji, & Jagirdar, 2003; Sakamaki et al., 2018), whereas ^1^H NMR spectrum of 1,3-dithiethane showed only one signal at *δ* 4.04 (s, 2H).

**Fig. 5:**
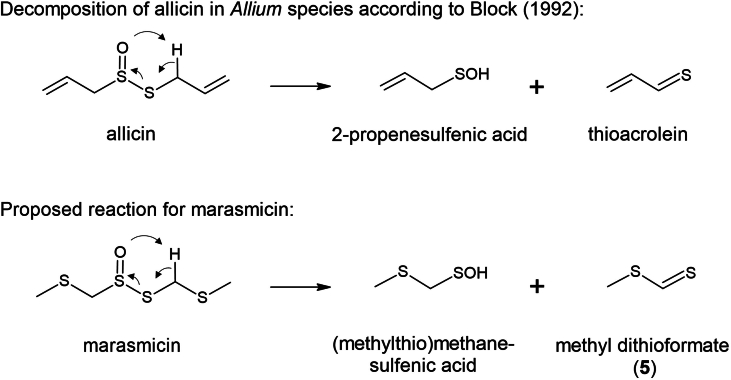
Possible decomposition pathways of the thiosulfinates allicin and marasmicin in garlic and *Mycetinis scorodonius* respectively.

A further indication that odorant no. 5 is methyl dithioformate is its possible origin from marasmicin (Fig. 5). According to Block, thioacrolein and 2-propenesulfenic acid are formed by Cope elimination from allicin in *Allium* species (Block, 1992). If this elimination is also applied to marasmicin, methyl dithioformate (5) and (methylthio)methanesulfenic acid are formed. The latter is very unstable and can be converted to methyl dithioformate by elimination of water. To verify that the main aroma compounds are not merely artifacts formed in the GC liner at 250 °C, cold-on-column injections were also performed using a GC-FID-O system. The two main aroma components of *M. scorodonius* (dichloromethane extract) methyl dithioformate (5) and 2,3,5-trithiahexane (11) could be detected both olfactorily and by comparison to an authentic standard. 2,4,5,7-Tetrathiaoctane (15) could be detected olfactorily. As a thioaldehyde, methyl dithioformate (5) is highly reactive and can be easily degraded.

### 3.4 VOCs and marasmicin in liquid extract

A dichloromethane extract of dried *M. scorodonius* fruiting bodies showed less peaks compared to the HS-SPME analysis: only four sulfur-containing VOCs were present, but no other peaks that could be attributed to the sample (Table 2). Methyl dithioformate (5) and 2,3,5-trithiahexane (11) were identified via standards. 2,4,5,7-Tetrathiaoctane (15) and 2,3,5,7-tetrathiaoctane 3,3-dioxide (16) were tentatively identified by MS. It would have been expected that the dioxide (16) is 2,4,5,7-tetrathiaoctane 2,2-dioxide, since it is derived from 2,4,5,7-tetrathiaoctane. However, a comparison with MS spectra from the literature showed that it is more likely to be 2,3,5,7-tetrathiaoctane 3,3-dioxide. MS spectra of 2,4,5,7-tetrathiaoctane (15) and 2,3,5,7-tetrathiaoctane 3,3-dioxide (16), as well as the reference spectra of 2,4,5,7-tetrathiaoctane 2,2-dioxide and 2,3,5,7-tetrathiaoctane 3,3-dioxide are shown in **Fig. S4** in the supporting information. 2,3,5,7-Tetrathiaoctane 3,3-dioxide (16) could not be detected in the HS-SPME samples.

**Table 2:** Volatile sulfur containing compounds found in the *Mycetinis scorodonius* dichloromethane extract, analyzed by GC–MS/MS-O (liquid injection, applied split ration of 20:1). Quantitation/semi-quantitation using internal standard diallyl disulfide. Identification by retention index (RI), mass spectrum (MS), olfactometry (O) and/or authentic standard (STD); na: not available.

compound	methyl dithioformate	2,3,5-trithiahexane	2,4,5,7-tetrathiaoctane	2,3,5,7-tetrathiaoctane 3,3-dioxide
compound no.	5^#^	11	15*	16*
perceived odor	garlic, sulfurous	aged garlic, savory, sulfurous	onion, savory, cabbage-like	cabbage-like, burned
odor from literature	garlic, sulfurous^1^	(long-standing) garlic, sulfurous, onion^2,3^	garlic, rotten, onion with irritating effect^1,3^	na
identification	RI, MS, O, STD	RI, MS, O, STD	RI, MS, O	RI, MS
RI polar (sample)	1253	1648	2264	2642
RI polar (literature)	1273^1^	1654^1^	2278^1^	na
RI nonpolar (sample)	< 800	1126	1513	1759
RI nonpolar (literature)	na	1139^4^	1528^5^	na
determined amount [mg/kg DM]	1008^sq^	631^q^	1699^sq^	680^sq^
odor threshold [*μ*g/kg]	na	40^3^	3.5^3^	na
odor activity value	na	15,779	485,478	na

^#^ identified within this work after synthesis of the compound; * tentatively identified; ^sq^ semi-quantitation; ^q^ quantitation; ^1^Sakamaki et al., 2018; ^2^Trapp et al., 2018; ^3^Kubota & Kobayashi, 1994; ^4^Rapior et al., 1997; ^5^NIST Chemistry WebBook, SRD 69 (2026b).

The same fungal dichloromethane extract used for GC analysis was also analyzed by UHPLC-HR-MS for the presence of the precursor marasmicin. The LC-MS spectrum showed a parent ion of [M + H]^+^ of 202.9685 *m/z* and a UV spectrum with a shoulder around 225 nm, which, according to Kubec et al. (Kubec et al., 2013), could correspond to marasmicin. The LC-MS spectrum was as follows: *m/z* (%) 92.9827 (100), 172.9579 (65), 124.9547 (54), 140.9859 (43) and 202.9685 (42) and 154.9652 (38). It is already known from the literature that marasmicin is present in the plant *T. violacea* (Kubec et al., 2013). Therefore, fresh leaves of the plant were also extracted and measured by UHPLC-HR-MS. The corresponding chromatograms are shown in the supporting information **Fig. S5** and **S6**. Both, the fungal and the plant extract showed a peak with [M + H]^+^ of 202.9685 *m/z* with highly similar MS- and UV-spectra (see supporting information **Fig. S7** and **S8**). Fragmentation in source (without elevated collision energies) generated fragments similar to the low-resolution fragmentation spectrum for marasmicin provided by Kubec et al. (MS/MS of parent ion 203 (positive mode), namely 203.2, 172.9, 141.0, 127.0 and 93.0) (Kubec et al., 2013).

Considering the literature reported presence of marasmicin in *T. violacea* plant extracts and the aforementioned results, the *T. violacea* extract was also analyzed via GC–MS/MS under the same conditions as the mushroom extracts. The chromatogram revealed the same four sulfur containing compounds as the fungal extract (methyl dithioformate (5), 2,3,5-trithiahexane (11), 2,4,5,7-tetrathiaoctane (15) and 2,3,5,7-tetrathiaoctane 3,3-dioxide (16)). These results allow to conclude that marasmicin is also present in the fungal extract.

### 3.5 GC–MS/MS analysis of UHPLC microfractions

It is often mentioned in the literature that sulfur-containing compounds, especially thiosulfinates, form artifacts at high temperatures, such as in GC (Block, 1992; Block, Putman, & Zhao, 1992). Due to the inherent instability of marasmicin, it has not yet been detected by GC in any publication (Gmelin et al., 1976; Kubec et al., 2013; Kubota & Kobayashi, 1994; Sakamaki et al., 2018). To investigate whether the aroma compounds found were merely artifacts, several analyses were performed in addition to the cold-on-column injections mentioned under 3.3: 1) The mushroom extract was analyzed for the presence of 2,3,5-trithiahexane (11) using UHPLC-DAD at 230 nm, and its existence was confirmed using an authentic standard (see supporting information **Fig. S9**). Since high temperatures were not used during sample preparation and separation, it can be concluded that 2,3,5-trithiahexane (11) is not only an artifact caused by high temperatures. It is more likely that it originates from the natural degradation of marasmicin itself, analogous to the release of aroma compounds from allicin in garlic. 2) In addition to the confirmation that 2,3,5-trithiahexane (11) is a naturally occurring degradation product, a total of 20 UHPLC-DAD runs were fractionated and combined. In addition to blank fractions, the marasmicin fraction F17 (identification of marasmicin see 3.4) and the 2,3,5-trithiahexane fraction F28 (see supporting information **Fig. S10**) were subsequently analyzed using GC–MS/MS, with remarkable results. No marasmicin itself was detected in the marasmicin fraction F17 using GC–MS/MS, but instead a mixture of methyl dithioformate (5), dimethyl trisulfide (8), 2,3,5-trithiahexane (11), 2,3,5-trithiahexane 2,2-dioxide, and 2,4,5,7-tetrathiaoctane (15; see supporting information **Fig. S11**). These results showed for the first time which degradation products pure marasmicin breaks down into when exposed to heat. Ranglová et al. conducted a stability study with marasmicin and showed that marasmicin was no longer detectable in water after 120 min at 80 °C (Ranglová et al., 2015). This verifies our result that marasmicin is indeed too unstable and can form the aforementioned degradation products in the GC system. It also explains why no marasmicin has been detected by GC to date.

In contrast, the 2,3,5-trithiahexane fraction F28 showed only two sulfur-containing substances in the GC system: a large 2,3,5-trithiahexane peak (11) and a small 2,3,5-trithiahexane 2,2-dioxide peak (see supporting information **Fig. S12**). 2,3,5-Trithiahexane (11) is stable enough to be analyzed by GC. 2,3,5-Trithiahexane (11) is therefore both a natural degradation product of marasmicin and also a thermally induced degradation product.

The results show that the aroma compounds found using GC (Table 1, Table 2) are attributable to both degradation products of marasmicin and heat-induced artifacts. Since *M. scorodonius* can also be used as a seasoning mushroom in hot dishes, the aroma compounds analyzed here can nevertheless play an important role and also partially reflect the aroma, especially 2,3,5-trithiahexane. Kubec et al. studied *T. violacea* and found many acyclic oligosulfides in the cooked rhizomes of the plant (Kubec et al., 2013). In comparison, they found none of these compounds in fresh, uncooked parts of the plant.

### 3.6 Quantitation of aroma compounds

Since it has now been shown that 2,3,5-trithiahexane is not only a thermally induced degradation product of marasmicin, it has also been quantitated. Quantitation of 2,3,5-trithiahexane (11) in the liquid extract yielded an amount of 631 mg/kg dry matter with a relative standard deviation (RSD) of 17%. Semi-quantitation of methyl dithioformate (5), 2,4,5,7-tetrathiaoctane (15), and 2,3,5,7-tetrathiaoctane 3,3-dioxide (16) yielded values of 1008, 1699, and 680 mg/kg, respectively, with RSDs of 5, 10, and 9%. Normally, the aroma content in mushrooms is in the *μ*g/kg range (Schmidberger & Schieberle, 2020; H. Zhang et al., 2018). Mushrooms such as *M. scorodonius* are therefore not called “seasoning mushrooms” without a reason. Kusterer et al. also found high levels of the precursor *γ*-glutamyl-marasmin in *M. alliaceus*, up to 2.99% in air-dried fruiting bodies (Kusterer et al., 2011). Garlic itself also contains high levels of allicin, about 3 g/kg (Rybak, Calvey, & Harnly, 2004; Z. Zhang et al., 2015). The signals detected are at least partially created by heat degradation during GC analysis. Concentrations are therefore not absolute and only fit to compare with other analysis performed with similar temperature parameters.

### 3.7 Determination of flavor dilution factors

**Fig. 6:**
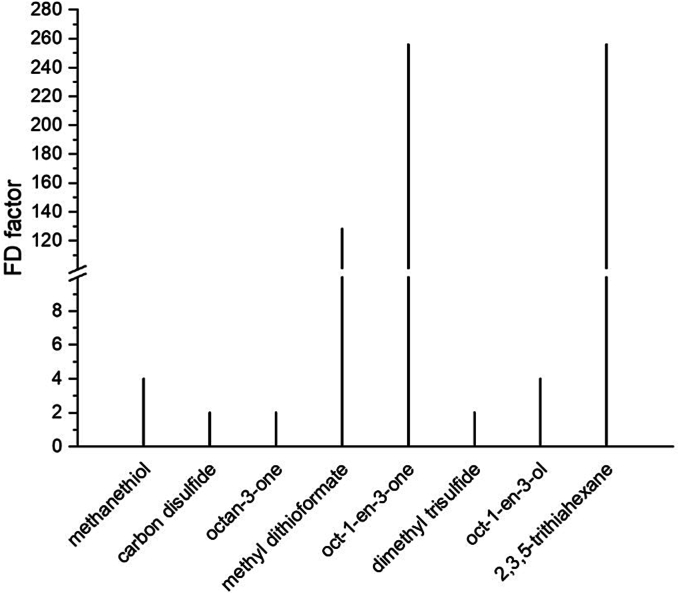
Median FD factors of the perceived aroma compounds of *M. scorodonius* (*n* = 3 people).

Among the aroma compounds presented in Table 1, only eight substances could be olfactorily perceived at the olfactory detection port using HS-SPME. For these compounds, the FD factors were determined (Fig. 6). Only two sulfur-containing substances had high FD factors: 2,3,5-trithiahexane (11; median FD 256) and methyl dithioformate (5; median FD 128). All three panelists were still able to detect 2,3,5-trithiahexane (11) at the highest dilution level. For methyl dithioformate (5), the individual measured values were FD 64, 128 and 256. The indication of the median FD does not show that the range of the individual measured values for dimethyl trisulfide (8) was considerable: FD 1, 2 and 64 (for further detailed values, see supporting information **Table S2**). Depending on the individual perception threshold of each person, dimethyl trisulfide (8) can therefore also contribute to the overall impression of the aroma. There is no available literature on the odor threshold of methyl dithioformate (5). It is assumed to be very low as sulfur-containing compounds are generally known to be highly potent (Marcinkowska & Jeleń, 2022). Methanethiol (1) and dimethyl disulfide (3) show values of 2.1 μg/kg and 8 μg/kg respectively (detected by electronic nose) (Deshmukh, Jana, Bhattacharyya, Bandyopadhyay, & Pandey, 2014). Kubota and Kobayashi determined the odor threshold values in water for 2,3,5-trithiahexane (11; 40 μg/kg) and 2,4,5,7-tetrathiaoctane (15; 3.5 μg/kg) (Kubota & Kobayashi, 1994).

### 3.8 Odor activity values

The odor activity value (OAV) is used to indicate the influence of an aroma compound on the aroma profile. It is defined as the ratio of the concentration of each compound to its odor threshold. Having the odor threshold value and the quantitated amount of 2,3,5-trithiahexane (11) in the extract it is possible to determine the OAV of 2,3,5-trithiahexane (11). Although the odor threshold with 40 *μ*g/kg (Kubota & Kobayashi, 1994), which was determined back in 1994, is quite high compared to other sulfur-containing substances, this still results in an OAV of 15,779 for 2,3,5-trithiahexane (11). The calculated OAV for the semi-quantitated 2,4,5,7-tetrathiaoctane is as high as 485,478 (odor threshold values in water of 3.5 *μ*g/kg (Kubota & Kobayashi, 1994)), with an odor impression at the ODP of onion, cabbage-like and savory (Table 2). OAVs in pan-fried shiitake mushrooms, for example, are much lower, ranging between 2 and 981 (Schmidberger & Schieberle, 2020). But when preparing the *Mycetinis* mushroom extract, the enzymatic reactions are activated by cell destruction and the addition of water. During the 30 min incubation period, a large proportion of the precursor *γ*-glutamyl-marasmin can be converted into marasmicin and further flavors. Therefore, in addition to high levels of 2,3,5-trithiahexane (11) and 2,4,5,7-tetrathiaoctane (15), for example, high OAVs are also feasible.

## 4 Conclusion

In summary, the work presented in this study has shown that the garlic-scented mushroom *Mycetinis scorodonius* can be cultivated under laboratory conditions and that the aroma profile of *M. scorodonius* is characterized by sulfur-containing substances derived from marasmicin. HPLC and GC measurements have shown which thermal degradation products can be formed from marasmicin. The main aroma compound, 2,3,5-trithiahexane, was quantitated and remains olfactorily detectable even at high dilutions, while methyl dithioformate was identified in mushrooms for the first time.

The instability of thiosulfinates such as marasmicin poses a major challenge in analytical studies. Therefore, the results are comparable only with similar analytical methods, a fact that should be considered.

For future research, the aim is to elucidate the biosynthetic pathway of marasmicin and its precursor, *γ*-glutamyl-marasmin. To date, there is only one publication, dating from 1976, that proposes the conversion of *γ*-glutamyl-marasmin to marasmicin (Gmelin et al., 1976). To the best of our knowledge, however, there has not yet been a single publication on the formation of *γ*-glutamyl-marasmin in fungi. The objective is to investigate whether l-cysteine is the precursor for the formation of *γ*-glutamyl-marasmin and which enzymes are involved in this process.

## CRediT authorship contribution statement

**Jenny Ahlborn:** Writing – original draft, Visualization, Validation, Supervision, Methodology, Investigation, Data curation, Conceptualization. **Deria Yusein:** Visualization, Investigation. **Christoph Hartwig:** Writing – review & editing, Visualization, Methodology, Investigation, Conceptualization. **Tatyana Zhuk:** Writing – review & editing, Methodology, Investigation. **Lea-Angel Emrich:** Visualization, Investigation. **Annika E.L. Beiderwieden:** Investigation. **Florian Birk:** Writing – review & editing, Investigation. **Andreas K. Hammer:** Writing – review & editing, Methodology. **Anne Steinkamp:** Writing – review & editing, Investigation.

## Ethical statements

The authors declare that, according to national guidelines, GC-O analysis are not subject to an approval process. Participants were not forced to participate, the study requirements and risks were known, and participants gave their verbal consent. Participant data was not disclosed without their knowledge, and they had the option to withdraw from the study at any time.

## Declaration of competing interest

The authors declare that they have no known competing financial interests or personal relationships that could have appeared to influence the work reported in this paper.

## Data Availability

Data will be made available on request.
