# Supplementary material for: Analysis and identification of volatile aroma compounds of the garlic-scented mushroom Mycetinis scorodonius
Source: Food Chem X. 2026 May 27;36:104033. doi: 10.1016/j.fochx.2026.104033 (PMC13241648; doi:10.1016/j.fochx.2026.104033)
Supplement: Supplementary file 1 — Supplementary material [file mmc1.docx]

**Supporting Information**

**Analysis and identification of volatile aroma compounds of the garlic-scented mushroom *Mycetinis scorodonius***

Jenny Ahlborn*^†^, Deria Yusein^†^, Christoph Hartwig^‡^, Tatyana Zhuk^†,§^, Lea-Angel Emrich^†^, Annika E. L. Beiderwieden^†^, Florian Birk^‡^, Andreas K. Hammer^‡^, Anne Steinkamp^‡^

^†^Institute of Food Chemistry and Food Biotechnology, Justus Liebig University Giessen, Heinrich-Buff-Ring 17, 35392 Giessen, Germany

^‡^Fraunhofer Institute for Molecular Biology and Applied Ecology (IME), Branch for Bioresources, Ohlebergsweg 12, 35392 Giessen, Germany

^§^Faculty of Chemical Technology, Igor Sikorsky Kyiv Polytechnic Institute, Beresteiskyi Ave, 37, Kyiv, Ukraine, 03056

*Corresponding author: Dr. Jenny Ahlborn. E-mail: jenny.ahlborn@lcb.chemie.uni-giessen.de. Phone: +49 641 9934928. Fax +49 641 9934909.

**Content of Supporting Information**

Page 3: **Figure S1:** Mass spectra of the two unidentified peaks (GC-MS/MS). Peak no. 12 with a molecular ion peak at *m/z* 124 (A) and peak no. 14 with a molecular ion peak at *m/z* 172 (B).

Page 3: **Figure S2**: Identification of odorant no. 5: The addition of the synthesized 1,3-dithiethane standard to a fruiting body extract shows no peak area increase of odorant no. 5 (A), but a new peak at 14.98 min (B) on a polar GC column.

Page 4: **Figure S3**: Identification of odorant no. 5: The addition of the synthesized methyl dithioformate standard to a fruiting body extract shows an increase in peak area (polar GC column).

Page 4: **Figure S4:** GC-MS/MS spectra of the tentatively identified substances 2,4,5,7-tetrathiaoctane (A) and 2,3,5,7-tetrathiaoctane 3,3-dioxide (B). Reference spectra of 2,4,5,7-tetrathiaoctane 2,2-dioxide (C; according to Kubota et al., 1994) and 2,3,5,7-tetrathiaoctane 3,3-dioxide (D; according to the NIST database Version 2.4). (B) and (D) match with a match factor of 906 and a probability of 97.8%.

Page 5: **Figure S5:** LC-MS Base peak chromatogram of full scan (red), UV-chromatogram (blue) and extracted ion chromatogram for putative marasmicin (202.9685±0.01) for plant sample (top) and fungal extract (bottom). Putative marasmicin at 5.1 min.

Page 5: **Figure S6:** Overlaid extracted ion chromatograms for putative marasmicin (202.9685±0.01). Plant extract in blue, fungal extract in black, putative marasmicin at 5.1 min.

Page 6: **Figure S7**: UV spectrum of the putative marasmicin peak (A) and full scan LC-MS spectrum (B) of the M. scorodonius mushroom extract. Injection volume of 1 µL, acquisition rate of 1 Hz.

Page 7: **Figure S8**: UV spectrum of the putative marasmicin peak (A) and full scan LC-MS spectrum (B) of the T. violacea plant extract. Injection volume of 0.1 µL, acquisition rate of 6 Hz.

Page 8: **Figure S9:** UHPLC-DAD chromatograms of M. scorodonius extract, 2,3,5-trithiahexane standard and spiked extract at 230 nm.

Page 8: **Figure S10:** Microfractions of 20 UHPLC runs (with 5 µL each) of the liquid M. scorodonius extract. Marasmicin fraction F17 at 4.9 min and 2,3,5-trithiahexane fraction F28 at 8.1 min. Fractions were collected in a 96-well plate.

Page 9: **Figure S11:** GC-MS/MS chromatogram of UHPLC fraction F17 (marasmicin) in comparison to the solvent blank. No marasmicin was found, but five degradation products occurred.

Page 9: **Figure S12:** GC-MS/MS chromatogram of UHPLC fraction F28 (2,3,5-trithiahexane) in comparison to the solvent blank. 2,3,5-Trithiahexane peak and a corresponding dioxide has been found.

Page 10: **Table S1**: Molecular masses and mass spectral data (m/z) for the aroma compounds analyzed in *M. scorodonius* using HS-SPME GC-MS/MS-O according to Table 1 in the publication.

Page 10: **Table S2**: FD factors (min, max, median) of *M. scorodonius* fruiting bodies analyzed by HS-SPME GC-MS/MS-O (n=3 people).

Page 11: **References**


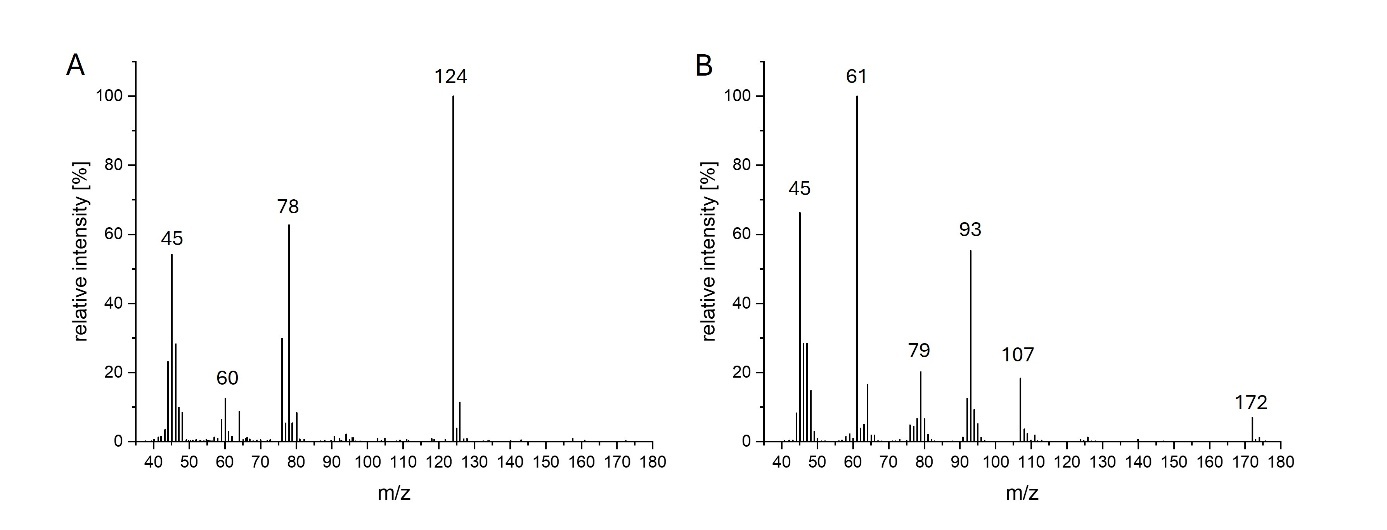


**Figure S1:** Mass spectra of the two unidentified peaks (GC-MS/MS). Peak no. 12 with a molecular ion peak at *m/z* 124 (A) and peak no. 14 with a molecular ion peak at *m/z* 172 (B).


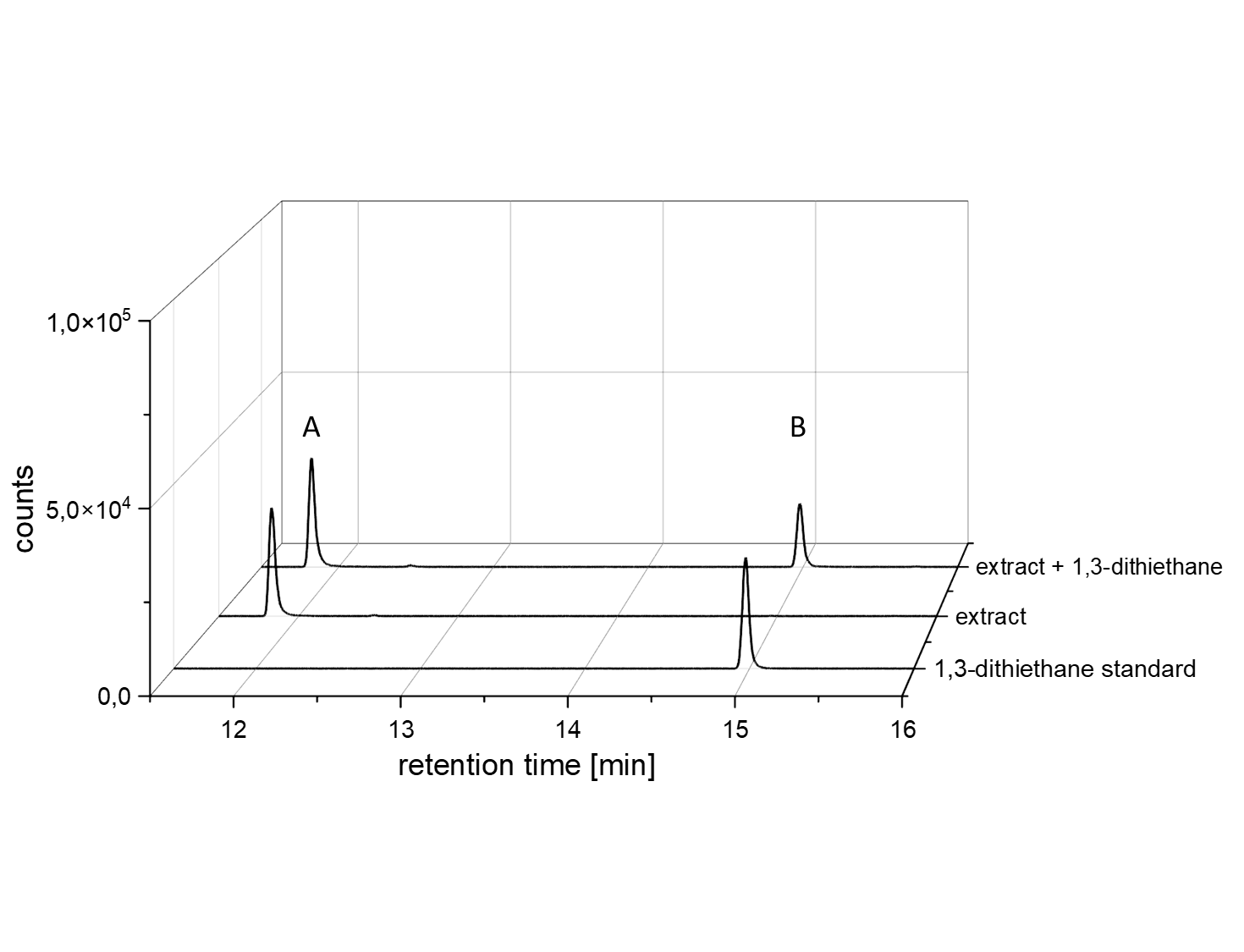


**Figure S2:** Identification of odorant no. 5: The addition of the synthesized 1,3-dithiethane standard to a fruiting body extract shows no peak area increase of odorant no. 5 (A), but a new peak at 14.98 min (B) on a polar GC column.


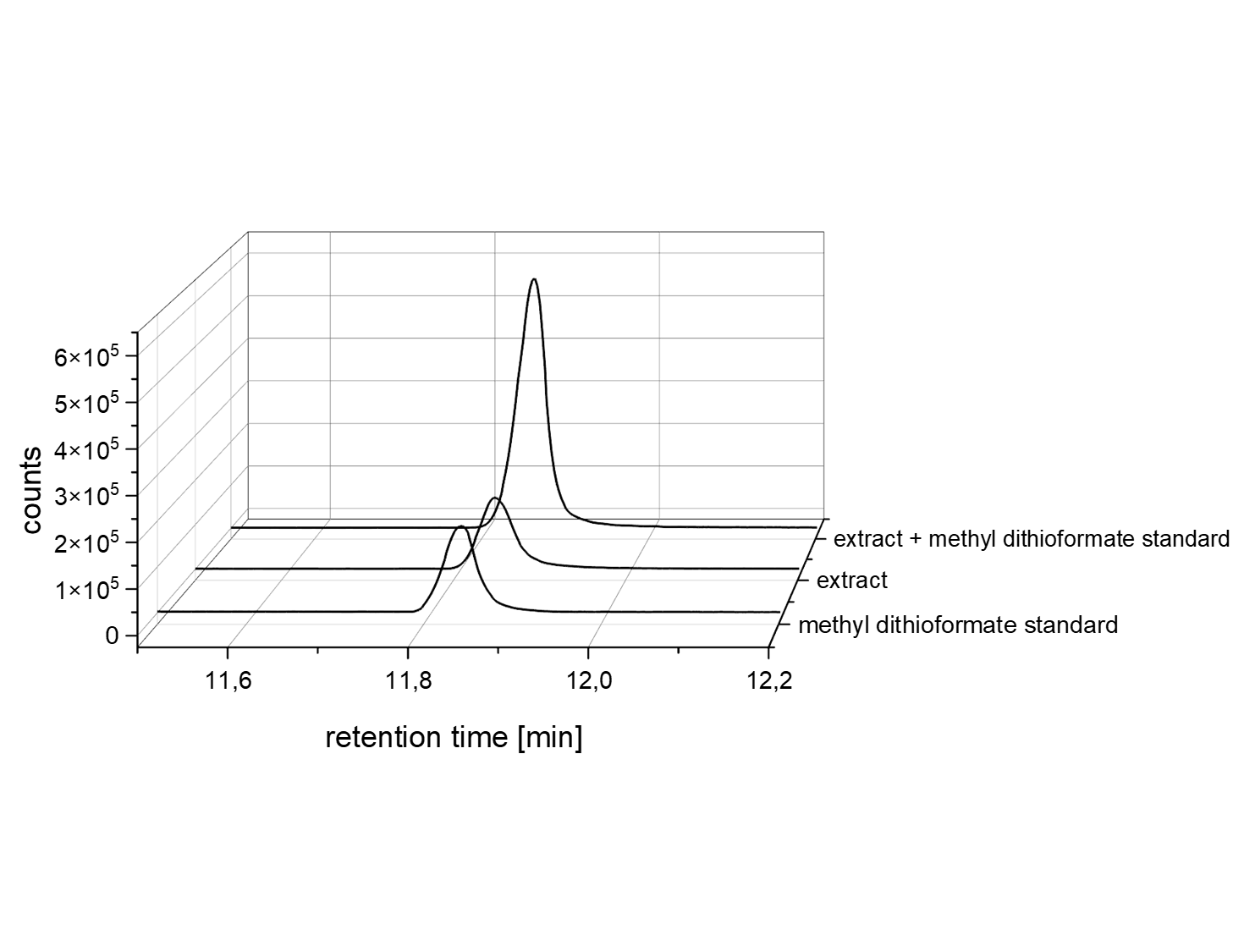


**Figure S3:** Identification of odorant no. 5: The addition of the synthesized methyl dithioformate standard to a fruiting body extract shows an increase in peak area (polar GC column).


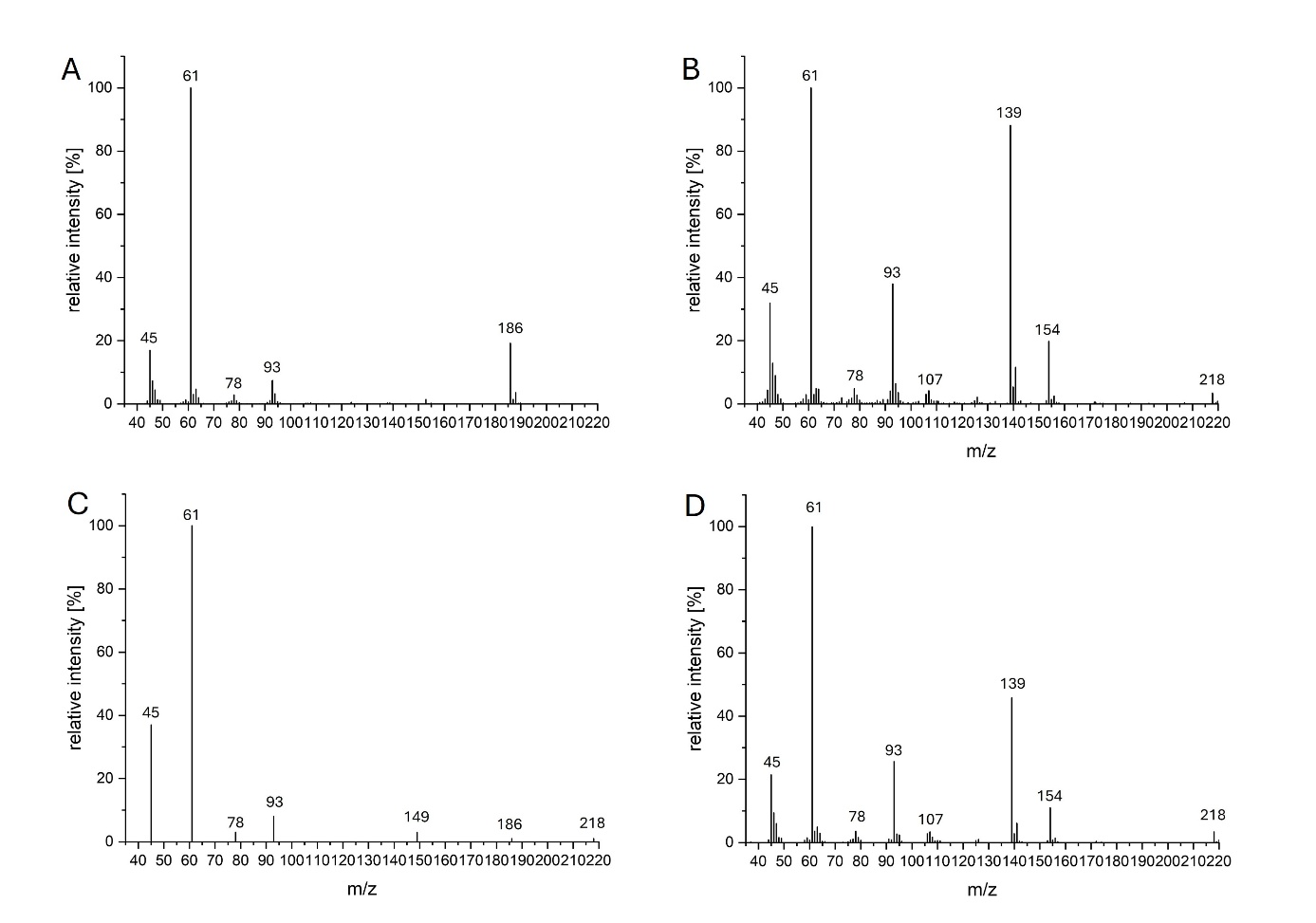


**Figure S4:** GC-MS/MS spectra of the tentatively identified substances 2,4,5,7-tetrathiaoctane (A) and 2,3,5,7-tetrathiaoctane 3,3-dioxide (B). Reference spectra of 2,4,5,7-tetrathiaoctane 2,2-dioxide (C; according to Kubota et al., 1994) and 2,3,5,7-tetrathiaoctane 3,3-dioxide (D; according to the NIST database Version 2.4). (B) and (D) match with a match factor of 906 and a probability of 97.8%.

**Figure S5:** LC-MS Base peak chromatogram of full scan (red), UV-chromatogram (blue) and extracted ion chromatogram for putative marasmicin (202.9685±0.01) for plant sample (top) and fungal extract (bottom). Putative marasmicin at 5.1 min.

**Figure S6:** Overlaid extracted ion chromatograms for putative marasmicin (202.9685±0.01). Plant extract in blue, fungal extract in black, putative marasmicin at 5.1 min.


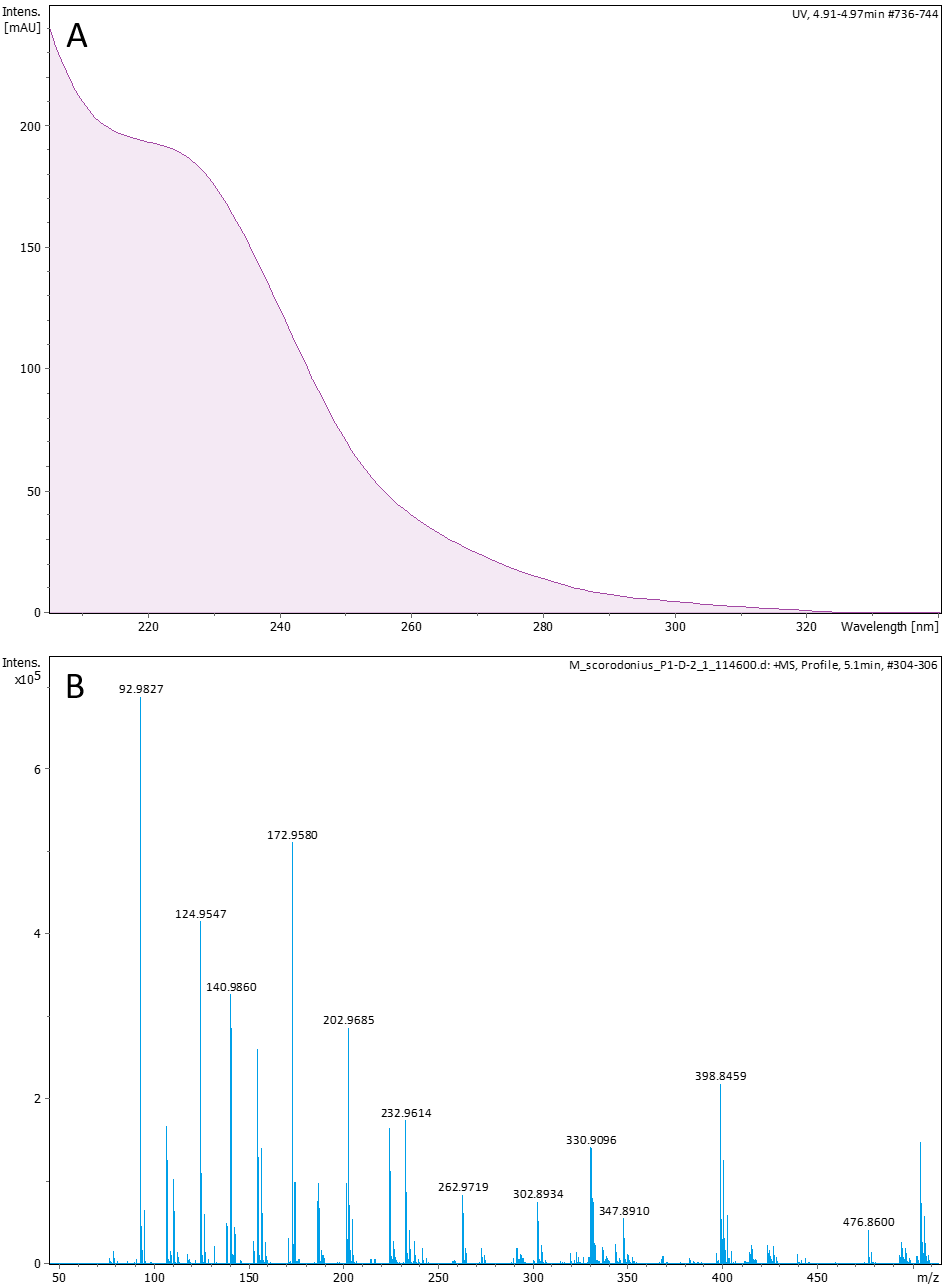


**Figure S7:** UV spectrum of the putative marasmicin peak (A) and full scan LC-MS spectrum (B) of the *M. scorodonius* mushroom extract. Injection volume of 1 *µ*L, acquisition rate of 1 Hz.


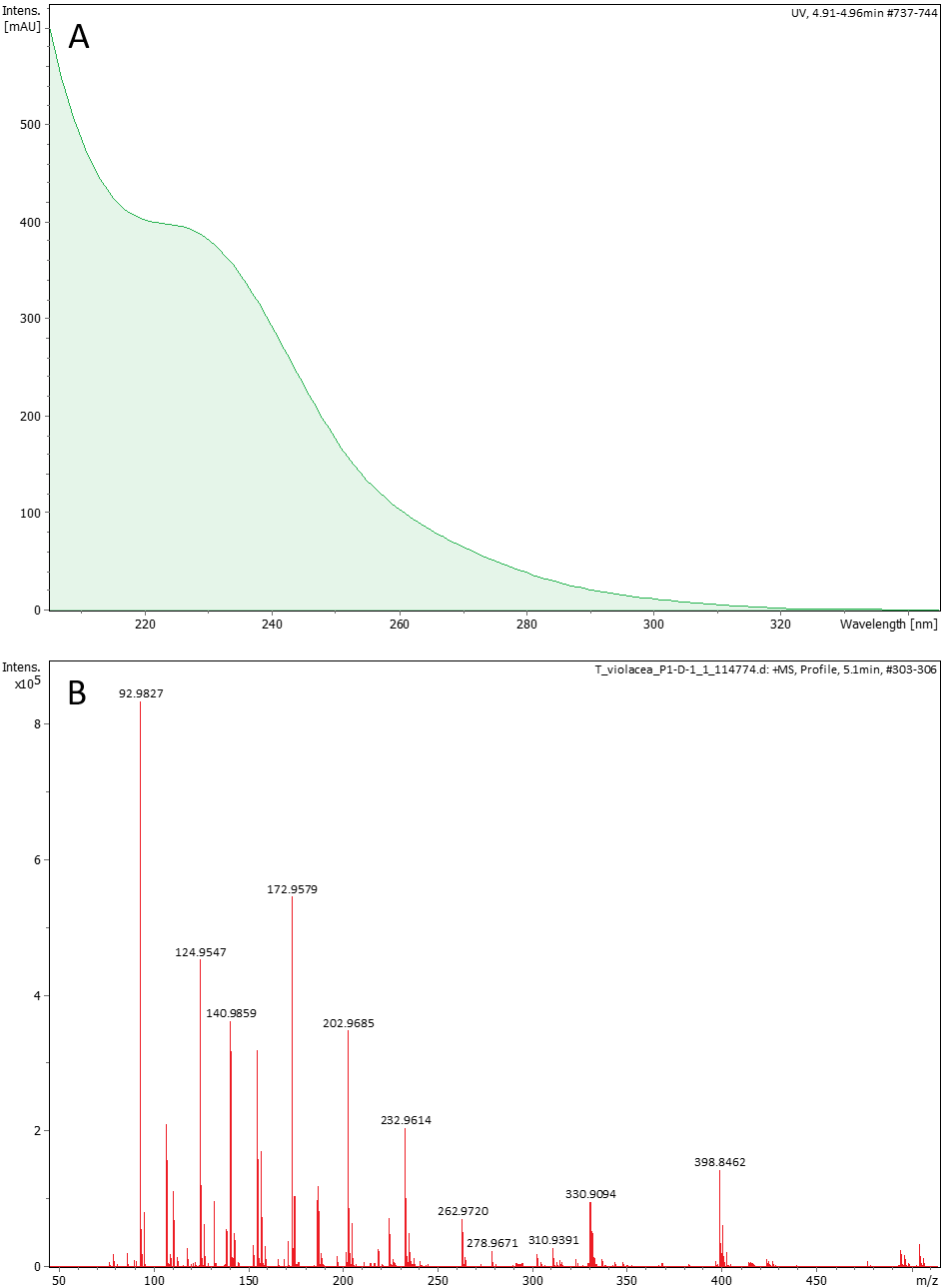


**Figure S8:** UV spectrum of the putative marasmicin peak (A) and full scan LC-MS spectrum (B) of the *T. violacea* plant extract. Injection volume of 0.1 *µ*L, acquisition rate of 6 Hz.


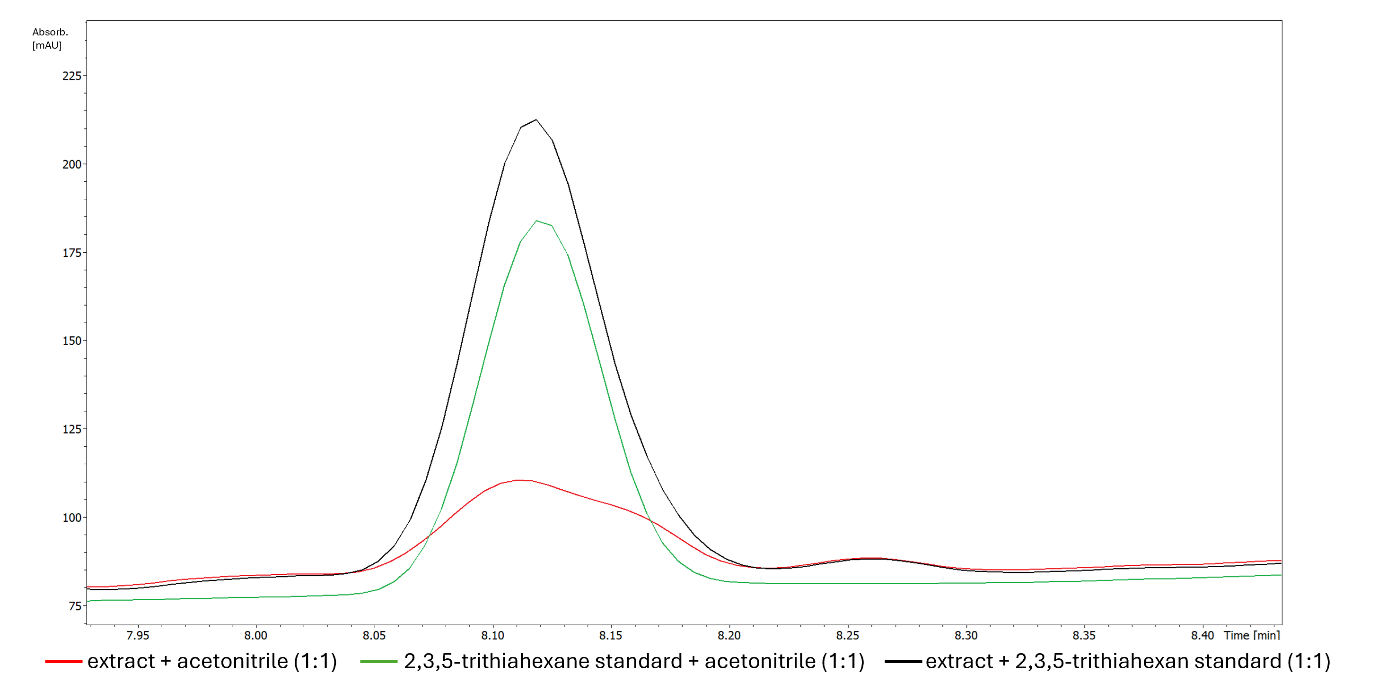


**Figure S9:** UHPLC-DAD chromatograms of *M. scorodonius* extract, 2,3,5-trithiahexane standard and spiked extract at 230 nm.


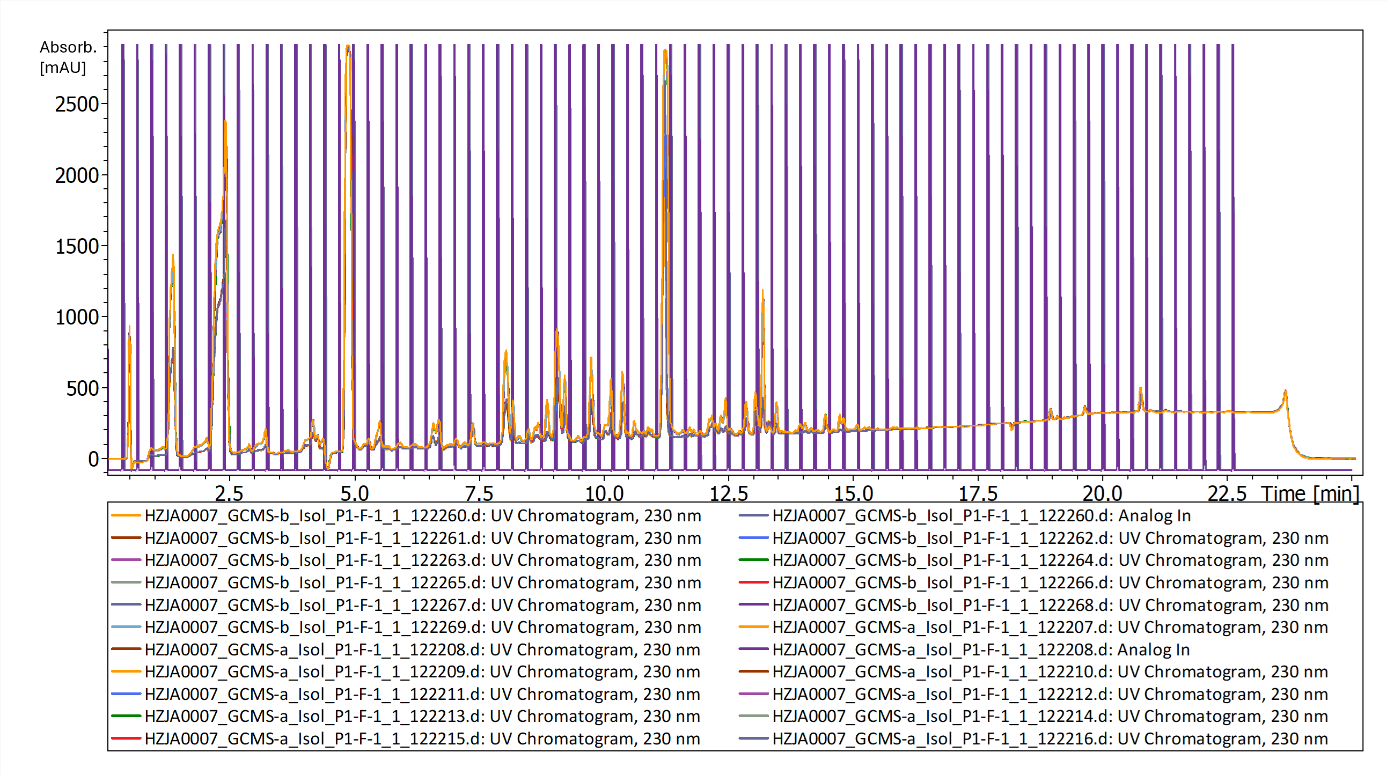


**Figure S10:** Microfractions of 20 UHPLC runs (with 5 µL each) of the liquid *M. scorodonius* extract. Marasmicin fraction F17 at 4.9 min and 2,3,5-trithiahexane fraction F28 at 8.1 min. Fractions were collected in a 96-well plate.


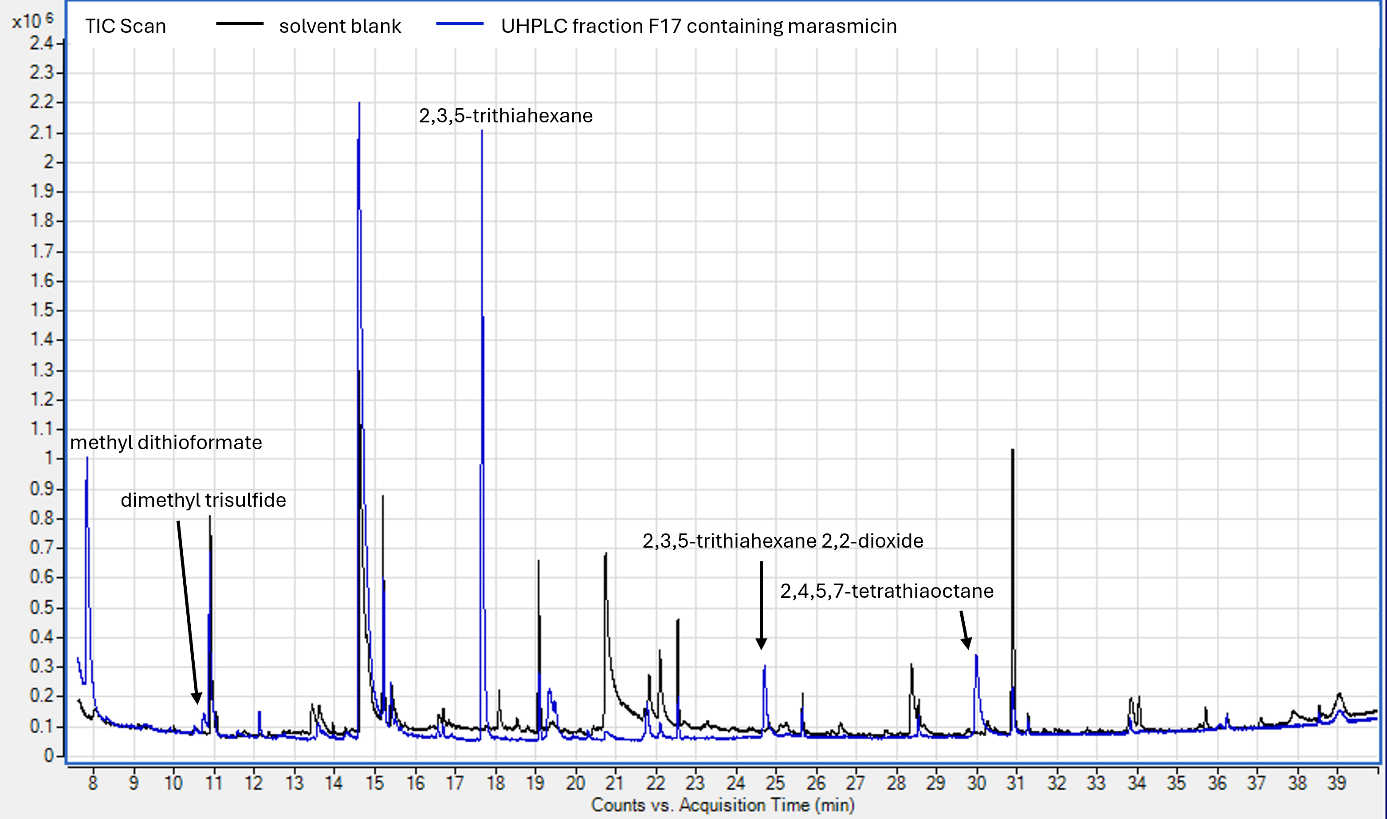


**Figure S11:** GC-MS/MS chromatogram of UHPLC fraction F17 (marasmicin) in comparison to the solvent blank. No marasmicin was found, but five degradation products occurred.


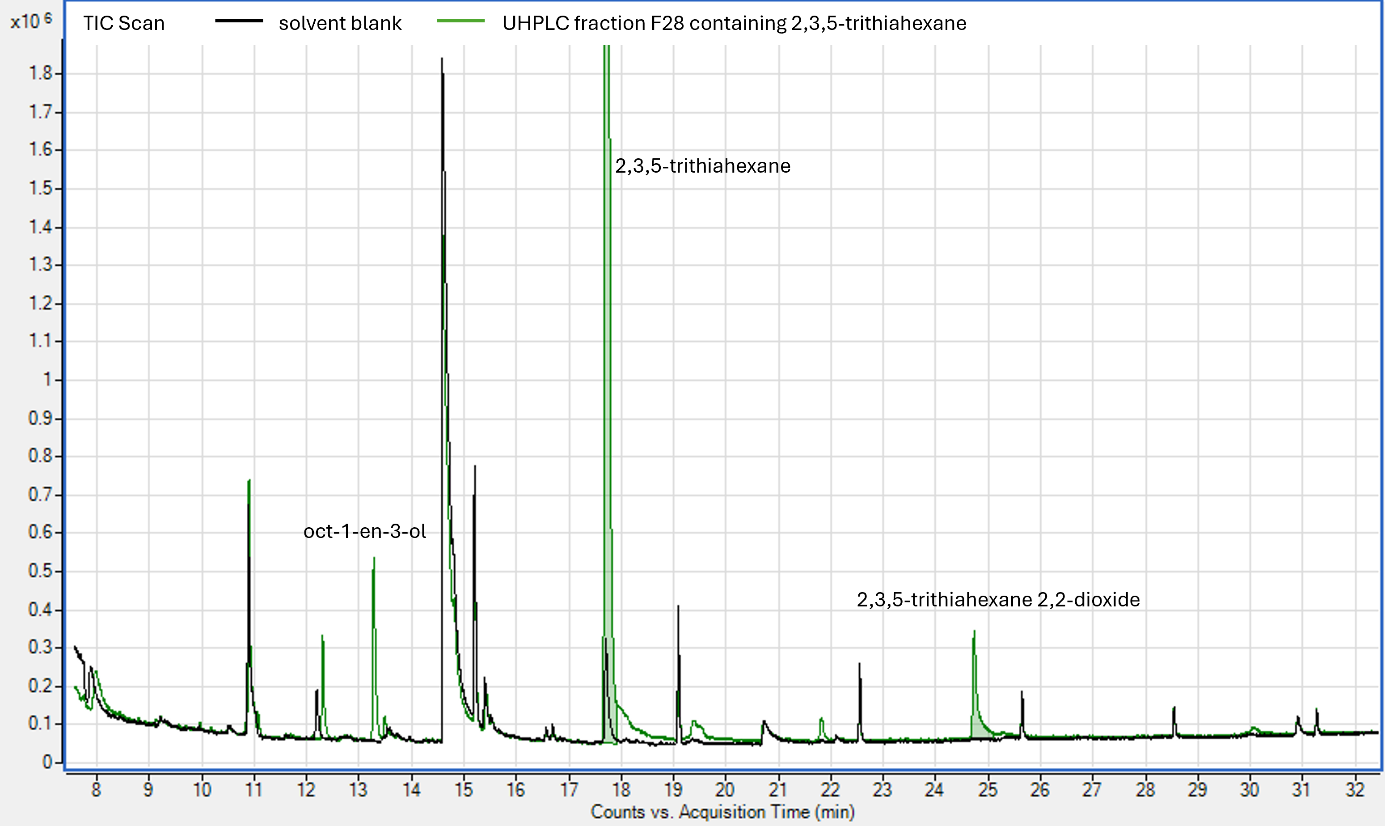


**Figure S12:** GC-MS/MS chromatogram of UHPLC fraction F28 (2,3,5-trithiahexane) in comparison to the solvent blank. 2,3,5-Trithiahexane peak and a corresponding dioxide has been found.

**Table S1:** Molecular masses and mass spectral data (*m/z*) for the aroma compounds analyzed in *M. scorodonius* using HS-SPME GC-MS/MS-O according to Table 1.

| no. | compound | molecular mass (g/mol) | mass spectral data, *m/z* (%) |
| --- | --- | --- | --- |
| 1 | methanethiol | 48.11 | 47 (100), 48 (87), 45 (59), 44 (52), 46 (16), 49 (6), 50 (4), 42 (3), 40 (2), 43 (2) |
| 2 | carbon disulfide | 76.14 | 76 (100), 44 (25), 78 (9), 77 (9), 48 (2), 45 (2), 63 (1), 64 (1), 47 (1), 43 (1) |
| 3 | dimethyl disulfide | 94.20 | 94 (100), 79 (44), 45 (31), 46 (18), 44 (18), 47 (16), 48 (10), 96 (10), 64 (9), 61 (8) |
| 4 | octan-3-one | 128.21 | 43 (100), 99 (94), 72 (86), 71 (75), 57 (60), 41 (22), 55 (17), 42 (16), 85 (15), 128 (12) |
| 5 | methyl dithioformate | 92.19 | 92 (100), 45 (31), 77 (17), 94 (10), 76 (7), 44 (5), 47 (5), 93 (4), 64 (4), 91 (4) |
| 6 | 2,4-dithiapentane | 108.23 | 108 (100), 61 (81), 45 (75), 44 (44), 92 (29), 46 (22), 48 (22), 47 (19), 43 (19), 76 (18) |
| 7 | oct-1-en-3-one | 126.20 | 70 (100), 55 (75), 97 (30), 43 (15), 83 (14), 71 (11), 41 (10), 42 (7), 56 (4), 111 (4) |
| 8 | dimethyl trisulfide | 126.26 | 126 (100), 79 (56), 45 (43), 64 (30), 47 (27), 46 (18), 80 (16), 111 (16), 128 (15), 44 (11) |
| 9 | octan-3-ol | 130.23 | 83 (100), 55 (86), 59 (82), 44 (71), 41 (59), 43 (46), 57 (37), 101 (36), 42 (31), 70 (26) |
| 10 | oct-1-en-3-ol | 128.22 | 57 (100), 72 (24), 43 (22), 55 (16), 41 (15), 85 (14), 67 (13), 81 (12), 68 (10), 99 (8) |
| 11 | 2,3,5-trithiahexane | 139.98 | 61 (100), 45 (36), 140 (21), 46 (16), 47 (10), 79 (9), 93 (5), 64 (5), 63 (5), 94 (5) |
| 12 | not identified |  | 124 (100), 78 (63), 45 (54), 76 (30), 46 (28), 44 (23), 60 (13), 126 (11), 47 (10), 64 (9) |
| 13 | dimethyl trithiocarbonate | 137.96 | 44 (100), 91 (76), 138 (59), 76 (37), 45 (35), 43 (27), 47 (21), 59 (14), 42 (13), 78 (12) |
| 14 | not identified |  | 61 (100), 45 (66), 93 (55), 47 (28), 46 (28), 79 (20), 107 (18), 64 (16), 48 (15), 92 (13) |
| 15 | 2,4,5,7-tetrathiaoctane | 185.97 | 61 (100), 45 (39), 46 (18), 47 (11), 186 (11), 94 (8), 93 (7), 92 (6), 44 (6), 48 (6) |

**Table S2:** FD factors (min, max, median) of *M. scorodonius* fruiting bodies analyzed by HS-SPME GC-MS/MS-O (n=3 people).

|  |  | FD factors | | |
| --- | --- | --- | --- | --- |
| no. | compound | min | max | median |
| 1 | methanethiol | 1 | 16 | 4 |
| 2 | carbon disulfide | 2 | 4 | 2 |
| 3 | dimethyl disulfide | 1 | 1 | 1 |
| 4 | octan-3-one | 1 | 4 | 2 |
| 5 | methyl dithioformate | 64 | 256 | 128 |
| 6 | 2,4-dithiapentane | 1 | 1 | 1 |
| 7 | oct-1-en-3-one | 256 | 256 | 256 |
| 8 | dimethyl trisulfide | 1 | 64 | 2 |
| 9 | octan-3-ol | 1 | 1 | 1 |
| 10 | oct-1-en-3-ol | 1 | 8 | 4 |
| 11 | 2,3,5-trithiahexane | 256 | 256 | 256 |
| 12 | dimethyl trithiocarbonate | 1 | 1 | 1 |
| 13 | 2,4,5,7-tetrathiaoctane | 1 | 1 | 1 |

**References**

Kubota, K., Matsumoto (Shimojima), M., Ueda, M., & Kobayashi, A. (1994). New Antimicrobial Compound from *Scorodocarpus borneensis* Becc. *Bioscience, Biotechnology, and Biochemistry*, *58*(2), 430–431. https://doi.org/10.1271/bbb.58.430
